# Dementia Prevention Self-Management in Older Thai Adults with Type 2 Diabetes: Development and Psychometric Properties of Two Questionnaires

**DOI:** 10.3390/nursrep14040277

**Published:** 2024-12-02

**Authors:** Noppamas Pipatpiboon, Jirapas Sripetchwandee, Eakachai Kantawong, Ruksanudt Budda, Daniel Bressington

**Affiliations:** 1 Department of Public Health, Faculty of Nursing, Chiang Mai University, Chiang Mai 50200, Thailand; duangduen.b@cmu.ac.th; 2 Neurophysiology Unit, Cardiac Electrophysiology Research and Training Center, Faculty of Medicine, Chiang Mai University, Chiang Mai 50200, Thailand; jirapas.sripetch@cmu.ac.th; 3 Department of Physiology, Faculty of Medicine, Chiang Mai University, Chiang Mai 50200, Thailand; 4 Faculty of Nursing, Praboromarajchanok Institute, Boromarajonani College of Nursing Chiang Mai, Ministry of Public Health, Chiang Mai 50180, Thailand; eakachai@bcnc.ac.th

**Keywords:** dementia preventive behaviors, diabetes, IFSMT, factor analysis, psychometric properties, nursing

## Abstract

Background/Objectives: The prevalence of dementia, a complication of uncontrolled type 2 diabetes (T2DM), is rising among older adults. Effective self-management for dementia prevention is essential, but no validated questionnaires currently exist to evaluate these behaviors. Methods: The Dementia Preventive Individual and Family Self-Management Process Questionnaire (DP-IFSM-PQ) and the Dementia Preventive Self-Management Behavior Questionnaire (DPSMBQ) were developed based on the Individual and Family Self-Management Theory to evaluate dementia prevention self-management behaviors in older adults with T2DM. Items for the DP-IFSM-PQ (30 items) and DPSMBQ (29 items) were generated through literature review and tested for face validity. A quantitative cross-sectional study evaluated their psychometric properties using exploratory factor analysis (EFA) (n = 311) and confirmatory factor analysis (CFA) (n = 254). Results: The final DP-IFSM-PQ comprises four factors and 29 items, showing acceptable fit with limited discriminant validity. The DPSMBQ includes seven factors and 27 items, demonstrating good fit and acceptable discriminant validity. Conclusions: The Thai-language DP-IFSM-PQ and DPSMBQ show reasonable psychometric properties for application in Thai older adults, but revisions of certain items and further studies are recommended to reassess their properties.

## 1. Introduction

Type 2 diabetes mellitus (T2DM) is a global public health concern, affecting 537 million people worldwide in 2021 [1], with projections to reach 783 million by 2045. Prevalence increases with age, peaking at 24% among individuals aged 70–79 [1]. Rising diabetes rates among older adults worldwide significantly impact healthcare costs and national economies [2]. This growing rate of T2DM among older adults has significant implications for healthcare systems, particularly due to the increased risk of complications such as dementia. Older adults with T2DM have double the risk of cognitive impairment compared to those without the condition [3].

T2DM-related dementia is linked to vascular disease, glucose toxicity, insulin changes, and amyloid plaque formation in the brain [3,4,5]. Dementia risk factors include high HbA1C levels, longer disease duration, smoking, alcohol use, and stress, while medication adherence, physical activity, and cognitive management are protective factors [6,7,8,9,10,11,12,13,14]. These findings imply that improving glycemic control and promoting cognitive and physical activities may reduce the risk of dementia from T2DM. Preventing dementia in T2DM patients is most effective when interventions are implemented early, particularly in the prodromal stage [3]. Therefore, it is advisable for older adults with T2DM to prevent the occurrence of dementia by adopting appropriate health behaviors during the earlier stages of the disease [15]. According to a comprehensive narrative review [16] on strategies for dementia prevention, which included randomized controlled trials (RCTs), systematic reviews, meta-analyses, and observational studies in humans and relevant work in animal models, it has been suggested that potential interventions designed to prevent cognitive decline should consist of self-management, involving problem-solving, decision-making, and effective resource utilization to improve health outcomes. The self-management aims to improve health behaviors, such as regular exercise and a healthy diet, also decrease vascular risk factors and reduce psychosocial stress [16].

A recent systematic review and meta-analysis of 44 randomized controlled trials (RCTs) and quasi-experimental studies involving 11,838 participants found that self-management is essential for adopting effective health behaviors to lower HbA1C levels and prevent complications from diabetes [17]. Additionally, family involvement plays a significant role in encouraging positive health behaviors. A systematic literature review [18] of ten qualitative studies with a total of 170 participants indicated that older adults often lack sufficient self-management and self-care knowledge. However, those who receive social support from family members exhibit improved self-management behaviors, suggesting that family engagement is crucial in promoting effective self-management among older adults [19].

Given the importance of family involvement in dementia prevention interventions, it is necessary to assess self-care behaviors in a family-inclusive manner. Although previous systematic reviews have identified instruments for measuring self-care behaviors in Type 2 Diabetes Mellitus (T2DM), none have specifically focused on dementia preventive behaviors in older adults [20]. The Individual and Family Self-Management Theory (IFSMT), developed by Ryan and Sawin [21], serves as a valuable framework for creating measures to evaluate dementia preventive behaviors in older adults with T2DM. This theory emphasizes the interconnectedness of individual and family roles in care, highlighting that changes in one family member can influence the entire family system.

The IFSMT comprises three key components: the contextual dimension, the process dimension, and the outcome dimension. The contextual dimension includes personal and family factors (such as attitudes, literacy, family structure), disease-specific factors (physiological changes and treatment complexity), and environmental factors (access to healthcare, neighborhoods, work, and culture). The process dimension involves knowledge and beliefs (health behaviors aligned with personal understanding), skills and self-control (goal-setting, decision-making, and self-reflection to improve behavior), and social facilitation (support and influence from family, peers, and medical professionals). The outcome dimension includes short-term outcomes (self-management behaviors related to risks, symptoms, and treatment) and long-term outcomes (the impact of achieving short-term goals) [21]. The theory suggests that contextual factors influence self-management participation, and improving the process dimension leads to more positive outcomes for individuals and families [21].

A recent integrative review found that the IFSMT framework has been applied in both qualitative and quantitative studies across various chronic and acute conditions, including HIV/AIDS in adolescents, chronic health conditions in youth, and non-alcoholic fatty liver disease in adults [22]. This review reported that the dimensions and categories of the IFSMT (and the interrelationships between these) were supported by the reviewed studies in a range of populations, including adolescents with HIV/AIDS, adolescents/young adults with chronic health conditions, and adults with non-alcoholic fatty liver disease [22]. However, these studies indicate that there is still a lack of empirical evidence regarding dementia prevention behaviors among older adults with T2DM.

The increasing aging population in Thailand underscores the importance of reducing T2DM risk in older adults. Thailand became a “complete-aged” society in 2022, with 20% of its population aged 60 and above, and is expected to become a “super-aged” society by 2036 [23]. In 2022, diabetes and metabolic syndrome were the second most common health issues among older adults [24]. Delaying the onset of complications, particularly dementia from diabetes, could improve quality of life and benefit families, society, and healthcare systems.

Given the need to measure dementia preventive behaviors and self-management processes in older adults with T2DM in a family-inclusive way—and the lack of suitable instruments capturing core elements of these phenomena—this study aimed to develop and validate two new questionnaires. These tools assess dementia preventive behaviors and the process dimension of dementia preventive self-management in older adults with T2DM, based on the IFSMT, in the context of Thailand.

## 2. Materials and Methods

### 2.1. Design

This study was divided into two phases to develop and validate two new measures, namely (1) the Dementia Prevention Individual and Family Self-Management Process Questionnaire (DP-IFSM-PQ) and (2) the Dementia Preventive Self-Management Behavior Questionnaire (DPSMBQ). In the first phase, item generation was developed by researchers with a literature review. The second phase was a quantitative cross-sectional study with a psychometric property questionnaire evaluation. This study was approved by the Research Ethics Committee, Faculty of Nursing, Chiang Mai University, No. 043/2566. All procedures contributing to this study were followed in accordance with the ethical standards outlined in the Declaration of Helsinki. The study was carried out from April to December 2023.

### 2.2. Participants and Recruitment

**Figure 1:**
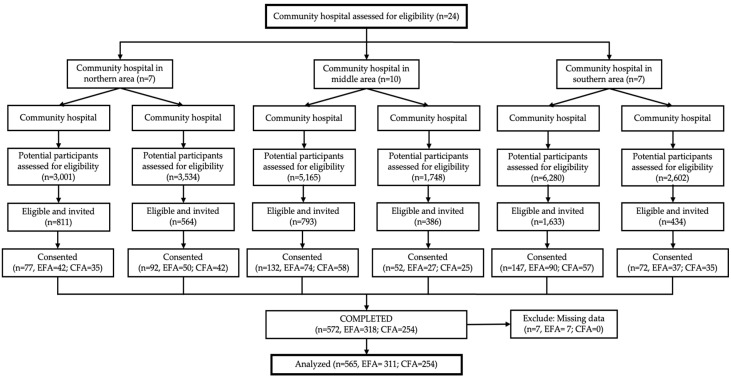
Participant flow diagram.

Older adults (aged 60–69 years old) with T2DM who were treated in a six-community hospital in Chiang Mai, Thailand, were enrolled in this study by multi-stage random sampling as shown in Figure 1. The inclusion criteria were (1) T2DM treated with medication or insulin; (2) living with family; (3) a baseline Instrument Activities of Daily Living Scale score of 16–20, indicating that they have the required ability to perform self-care activities at an advanced level; (4) able to read, write and communicate in Thai; (5) no visual or severe hearing problems; and (6) understand the study procedures and agreed to participate by signing the consent form. The exclusion criteria were (1) a diagnosis of depression, stroke, or Parkinson’s disease; (2) a history (or family history) of dementia, brain infection, brain injury, or psychiatric illness; and (3) a baseline Thai Mental State Examination (TMSE) score of at least 23, indicating dementia.

Participants were recruited by a trained research assistant in the outpatient’s department of the hospital when they attended their regular T2DM follow-up appointments. The research assistant had no role in the clinical care of potential participants. All study participants provided their written informed consent, and they were aware of the voluntary nature of taking part.

### 2.3. Item Generation

#### 2.3.1. The Dementia Preventive Individual and Family Self-Management Process Questionnaire (DP-IFSM-PQ)

The item categories were developed from a comprehensive review of dementia prevention studies based on the process dimension of the IFSMT. Literature published between 2009 and 2022 was searched in five databases: PubMed, Google Scholar, Scopus, Science Direct, and Thai Journals Online. A combination of search terms was used along with relevant Boolean operators, including ‘Individual and Family Self-Management Theory, Diabetes Mellitus, Dementia, self-management, elderly/older adult’. Finally, three components, namely knowledge and beliefs, self-regulation skills and abilities, and social facilitation, were generated. The contents were divided into 3 factors (30 items), including (1) the knowledge and beliefs of dementia prevention (15 items), (2) self-regulation skills, the ability to carry out dementia prevention behaviors (12 items), and (3) the social facilitation necessary to carry out dementia prevention behaviors (3 items). A 4-point Likert scale (1 = strongly disagree, 2 = disagree, 3 = agree, and 4 = strongly agree) was used. This questionnaire was developed using entirely positive questions, since these questions can foster a constructive and empowering assessment process of self-management that focuses on dementia prevention behaviors [25].

#### 2.3.2. The Dementia Preventive Self-Management Behavior Questionnaire (DPSMBQ)

The item categories were modified from our previous study [26] and a review of published work was carried out from 2000 to 2022 on dementia prevention self-management behaviors among older adults with T2DM in 5 databases (PubMed, Google Scholar, Scopus, Science Direct, Thai Journals Online). The following key words were used: Diabetes Mellitus, Dementia, self-management, and elderly/older adult. The items categories were finally selected from four articles [26,27,28,29].

The data were subjected to content analysis to categorize behaviors related to dementia prevention. Self-management behaviors concerning dementia prevention were classified into 6 factors (29 items), including:

- Dietary habits (13 items, item 1–13);
- Non-smoking and alcohol-avoiding habits (3 items, item 14–16);
- Leisure and exercise habits (3 items, item 17–19);
- Stress management and brain exercise (3 items, item 20–22);
- Depressant prevention behavior (3 items, item 23–25);
- Drug adherence and follow-up habits (4 items, item 26–29).

A 4-point Likert scale was utilized for 17 items measuring positive behaviors (item 1–3, 5–6, 16–26, and 29), with 1 representing “never”, 2 representing “sometimes”, 3 representing “often”, and 4 representing “always”. Conversely, this Likert scale was reversed (1 = always, 2 = often, 3 = sometimes, and 4 = never) for use in 12 items measuring negative behaviors (item 4, 7–15, and 27–28). The questionnaire was designed with both positive and negative questions to minimize response bias.

### 2.4. The Demographics Questionnaire

The demographics questionnaire included items on age, gender, marital status, educational level, employment, family members, family monthly income, family members with T2DM, duration of diabetes, and HbA1C level.

### 2.5. Assessment of Content Validity Indexes—DP-IFSM-PQ and DPSMBQ

Content validity indexes were calculated for each item using the I-CVI, with the number of experts giving a rating of “very relevant” in 4-point Likert-type scale for each item divided by the total number of experts. An I-CVI score of 0.79 or higher was considered appropriate [30]. The overall scales’ content validity was calculated using S-CVI/Ave where all items’ I-CVI was summed and divided by the total number of items (with an S-CVI/Ave ≥  0.9 indicating excellent content validity) [30]. The DP-IFSM-PQ and DPSMBQ CVIs are shown in Tables S1 and S2. The determination was carried out by six experts individually, including (1) a clinical instructor who specializes in self-management behaviors regarding dementia prevention among older adults with T2DM, (2) two nursing instructors who specialize in self-management behaviors regarding dementia prevention among older adults with T2DM, (3) a nursing instructor who specializes in the development of research tools, and (5) two nursing instructors who specialize in community-based nursing.

After reviewing the statements following experts’ suggestions, the face validity was evaluated by 10 older adults with T2DM. The participants read each question, and the researchers asked them to evaluate each statement by commenting on clarity, ease of use, and appropriateness. Based on their comments, no additional items were required for the DP-IFSM-PQ, as participants reported sufficient understanding. In contrast, more details were added to seven items of the DPSMBQ to increase their clarity, such as providing examples of foods that are high in sugar (item 7), foods that have high sodium (item 10), seasonings (item 11), foods that are high in saturated fat (item 12), foods that are high in cholesterol (item 13), types of physical movements (item 17), and types of moderate-level activities/exercises (item 18).

### 2.6. Psychometric Property Evaluation

#### Construct Validity

A cross-sectional study was carried out to test construct validity. First, we evaluated the exploratory factor analysis (EFA) with a varimax between April to July 2023. The Kaiser–Meyer–Olkin (KMO) and Bartlett’s test of sphericity were used to determine the appropriate sample size for factor analysis. Eigenvalues greater than or equal to 1.0 and factor loading graters greater than 0.5 were considered appropriate to verify the possible underlying factors. The internal consistency reliability was measured by Cronbach’s alpha coefficient with 30 participants. A Cronbach’s alpha of 0.65–0.80 is often considered “adequate” and therefore acceptable for internal consistency [31,32].

The sample size for EFA was determined by choosing ten people per item of the instrument (10 × 30 = 300) [32]; a sample size of 300 was required. Thus, our 311 participants were considered an adequate sample size. The confirmatory factor analysis (CFA) was performed with M+ between September–December 2023. The recommended sample size for CFA is suggested to be more than 150 participants [33]. While 150 participants met the minimum sample-size requirements, we planned to collect as many samples as possible during the data-collection phase. This resulted in a larger sample size (254), potentially increasing statistical power and precision. The goodness-of-fit indices were calculated by using the global fit of the model. The indices included chi-square ratio with degrees of freedom (χ^2^/df), root mean square error of approximation (RMSEA), comparative fit index (CFI), and Tucker–Lewis index (TLI). A χ^2^/df value 0 ≤ χ^2^/df ≤ 2 shows a good fit, while 2 < χ^2^/df ≤ 3 shows an acceptable fit. A CFI and TLI value 0.97 ≤ CFI ≤ 1.00 show a good fit of data, while 0.95 ≤ CFI < 0.97 shows an acceptable fit of data. A RMSEA value 0 ≤ RMSEA ≤ 0.05 can be considered as a good fit, and 0.05 < RMSEA ≤ 0.08 can be considered as an acceptable fit [34]. After evaluating the model fit, construct reliability (CR) for convergent validity and average variance reliability extracted (AVE) for discriminant validity was performed.

### 2.7. Data Analysis

Data were analyzed using IBM SPSS v26.0 and Mplus v8.6 with the maximum likelihood with a robust standard errors (MLR) estimator. Descriptive statistics were used for demographic data. The structural validity was investigated using exploratory factor analysis (EFA), confirmatory factor analysis (CFA), and internal reliability with Cronbach’s alpha coefficient. Also, data met the requirements of normality, linearity, and multicollinearity. *p*-values of <0.05 were considered statistically significant. Missing data were managed by listwise deletion (i.e., the entire case was not included in the analyses if there was a missing variable/question item).

## 3. Results

A total of 22,330 medical records were screened and 4621 older adults were identified that met the study inclusion criteria (i.e., 20.60% were found eligible). The research assistant invited all of those eligible to join the study based on the sampling strategy (see Figure 1). Finally, 572 of the 4621 invited older adults completed the survey, demonstrating a response rate of 12.38%. Of these responses, 7 were incomplete due to some missing responses and were therefore not analyzed, resulting in 565 completed surveys being included in the analyses.

### 3.1. Participant Demographics

The sociodemographic data acquired for EFA demonstrated that average age of participants was 64.85 (±2.77) years, with the majority being female (62.38%) and married (75.88%). Most participants had completed elementary school (71.06%) and most participants were either laborers (42.44%) or unemployed (35.69%). The majority of the participants’ family consisted of three people with extended family (41.80%). Most family members had no T2DM (72.03%). Most of them were Buddhist (97.43%). The average duration of T2DM in these participants was 10 years with a mean HbA1C of 7.67 mg/dL. Regarding the sociodemographic characteristics of participants for the CFA, two-thirds of participants were female (65.35%) with a mean age of 64.43 years, and most were married (75.59%). Consistent with data from the EFA, most participants had completed primary school (73.23%) and were laborers (49.21%) or unemployed (32.68%). Also, most of the participants’ family in CFA consisted of three persons and almost half of them were extended family (45.28%). Almost 70% of family members had no T2DM (69.69%). However, they had a mean monthly income of less than USD 300. Most participants were Buddhist (97.64%). The duration of T2DM in these participants was 9.8 years with a 7.64 mg/dL of average HbA1C level. The combined sociodemographic data from the EFA and CFA are shown in Table 1.

### 3.2. Principle Component Analysis

The Kaiser–Meyer–Olkin (KMO) analysis was applied to examine the factor structure. The DP-IFSM-PQ KMO index was 0.958 and Bartlett’s test of sphericity was X2 = 10,946; *p* = 0.00. The DPSMBQ KMO index was 0.865 and Bartlett’s test of sphericity was X2 = 4378; *p* = 0.00. These results have demonstrated that our data from two questionnaires constitute sufficient samples for PCA analysis.

#### 3.2.1. DP-IFSM-PQ: EFA

The exploratory factor analysis of 30 items of DP-IFSM-PQ was performed using principal component extraction and varimax rotation. The initial factor analysis of DP-IFSM-PQ showed a four-factor model (76.398%) with 29 original items included (item 11 was excluded due to factor loading less than 0.50). Table 2 shows the loading factor of each item after the varimax rotation. According to internal consistency analyses using in DP-IFSM-PQ, all extracted factors in both questionnaires have good to excellent internal reliability (Cronbach’s α= 0.855–0.927).

#### 3.2.2. DPSMBQ: EFA

The initial factor analysis (29 items) identified a seven-factor model explaining 65.399% of the variance. However, items 10 and 12 did not align with any factor, as their factor loadings were below 0.50, suggesting possible cross-loading onto another factor. Therefore, the original 27 items were retained in the questionnaire. Table 3 presents the factor loadings for each item following varimax rotation. Based on an internal consistency analysis for DPSMBQ, all factors in both questionnaires demonstrated good to excellent reliability (Cronbach’s α = 0.805–0.905).

### 3.3. Confirmatory Factor Analysis

#### 3.3.1. DP-IFSM-PQ: CFA

**Figure 2:**
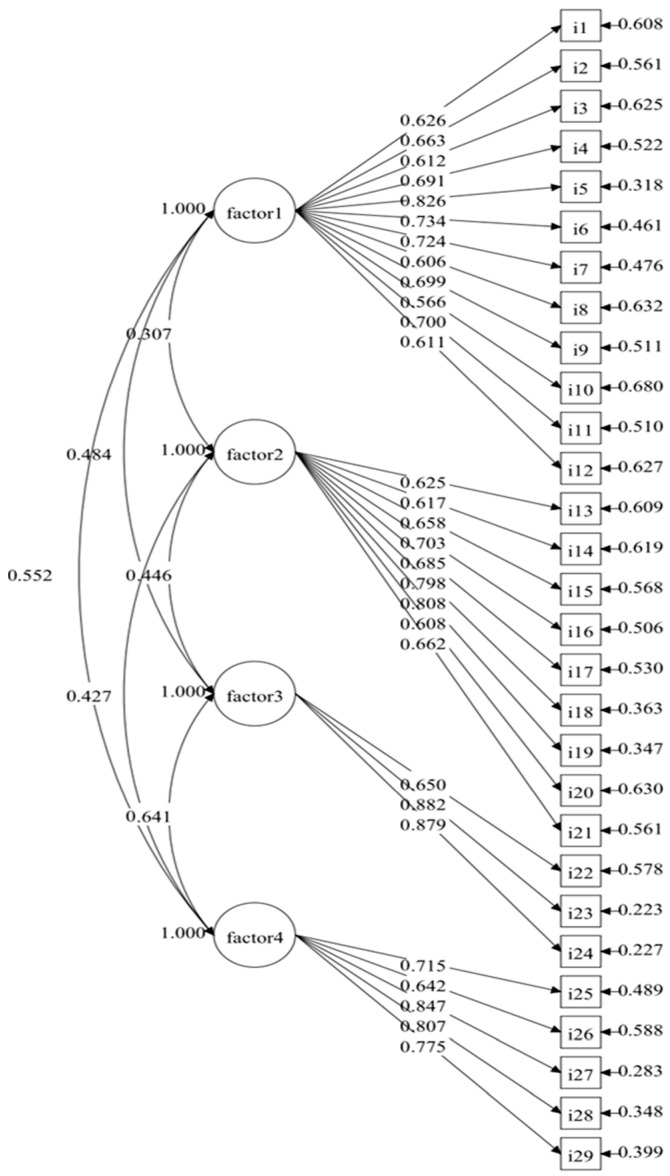
The results of the CFA of the DP-IFSM-PQ.

During the confirmatory factor analysis (CFA) of the DP-IFSM-PQ, the goodness of the proposed 4-factor model was evaluated using a range of fit indices, including the χ^2^/df ratio, root mean square error of approximation (RMSEA), comparative fit index (CFI), and Tucker–Lewis’s index (TLI). The results from the CFA indicated an acceptable fit of the measurement model to the data (χ^2^/df = 2.094, RMSEA = 0.066, CFI = 0.868, TLI = 0.855). Figure 2 illustrates that most factor loadings were above 0.600 (ranging from 0.608 to 0.882), except for item 10 in factor 1, which had a loading lower than the recommended value (λ = 0.566). In addition, the composite reliability (CR) for each factor, which exceeded the acceptable level of 0.60 (ranging from 0.850 to 0.942), indicated the presence of convergent validity. Additionally, the average variance extracted (AVE), noted in Table 4, was computed to assess the discriminant validity of this proposed 4-factor model. The AVE of factor 3 (0.658) and factor 4 (0.578) exceeded 0.500, while those of factors 1 and 2 were slightly below the recommended threshold. Similarly, the AVE of factor 1 was slightly smaller than some correlation coefficients between each of those factors and another factor, demonstrating limited discriminant validity.

#### 3.3.2. DPSMBQ: CFA

**Figure 3:**
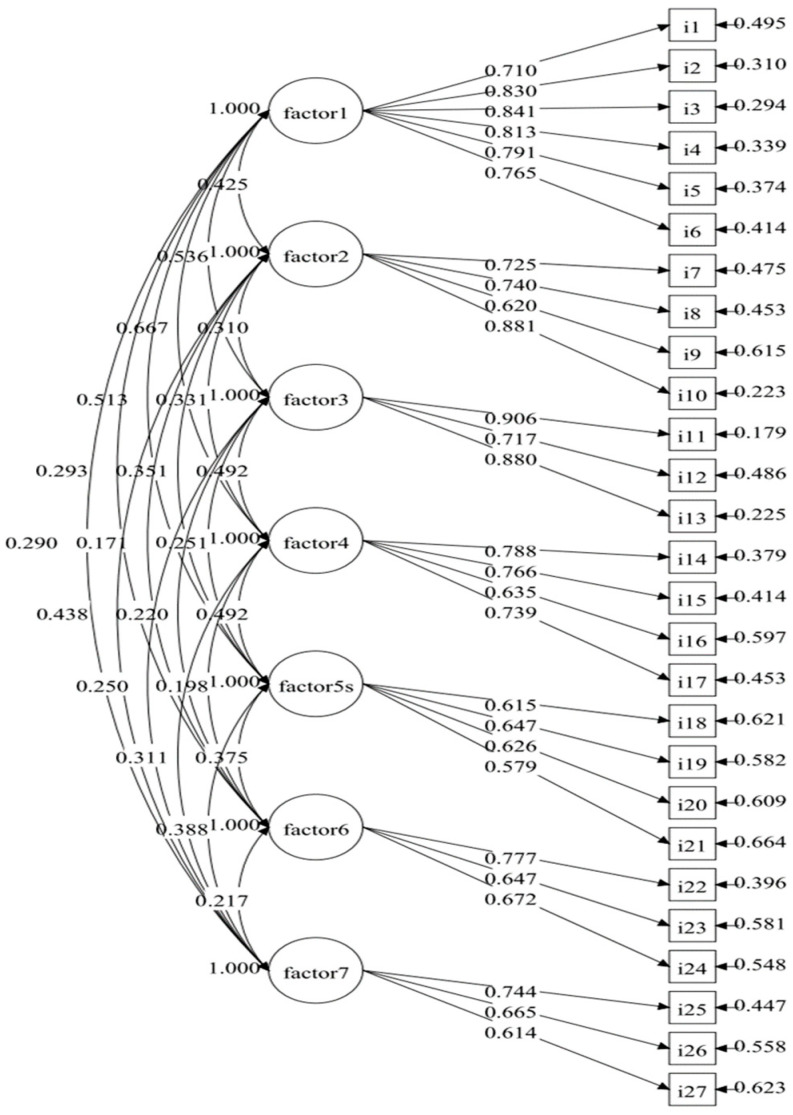
The results of the CFA of the DPSMBQ.

For the DPSMBQ, CFA was employed to assess the goodness of the measurement model using a range of global fit indices (χ^2^/df ratio, RMSEA, CFI, and TLI). The results indicated that the initially proposed correlated 7-factor model demonstrated a good fit across all employed fit indices (χ^2^/df = 1.828, RMSEA = 0.057, CFI = 0.898, TLI = 0.882). Most factor loadings for indicators were above the recommended threshold of 0.600, ranging from 0.614 to 0.906, with statistical significance (*p* < 0.001) as depicted in Figure 3. However, the factor loading of item 21 within factor 5 fell slightly below this threshold. Furthermore, the CR for each factor (ranging from 0.711 to 0.910) exceeded the acceptable level of 0.700, indicating relatively good convergent validity [34]. In Table 5, the AVE for factors 3, 4, and 5 was slightly lower, while for most factors, the AVE surpassed the acceptable value of 0.500 (ranging from 0.539 to 0.703). Moreover, each factor’s AVE was higher than any correlation coefficients between that factor and another, which reflects acceptable discriminant validity.

## 4. Discussion

This study aimed to develop and evaluate the properties of two new questionnaires for Thai older adults with T2DM, the DP-IFSM-PQ, and the DPSMBQ. According to the face validity testing, the statements of items in both questionnaires were shown to be well understood in terms of suitability, cultural, and social appropriateness.

According to the theoretical framework, the DP-IFSM-PQ should consist of three factors [21]. However, following the EFA measurement, four factors were found, three of which matched the theoretical framework: knowledge and beliefs, self-regulation skills and abilities, and social facilitation. Furthermore, self-efficacy was separated from the knowledge and belief factor. The reason for the differences between the EFA results and the originally hypothesized numbers of factors may be due to the participants’ comprehension of knowledge and beliefs as differing from self-efficacy. The current study’s results demonstrate that the process of self-management factors is consistent with Areri et al.’s study findings, which propose measuring four factors including knowledge, self-efficacy, self-regulation abilities, and social facilitation [35]. Some earlier studies which have used the IFSMT to develop questionnaires report a three-factor model [36,37]; however, these studies only assessed construct validity rather than CFA.

In addition, the CFA in this model was found to be an acceptable fit, which differs from an earlier study on an instrument for HIV self-management in adolescents using the IFSMT [38], which resulted in a model that supports the structural validity with a better fit than the current study. Since this is the first study to evaluate the psychometric properties of the questionnaires for older adults using the IFSMT framework, it is possible that the age and educational levels of participants may influence the model fit of the questionnaire [39]. Furthermore, construct validity has been shown to vary by age [40]. Also, we found that the AVE of factors 1 and 2 were less than 0.5. Although these values were within an acceptable range [41], we suggest providing additional information for each item to enhance participants’ comprehension. Additionally, the AVE of factor 1 demonstrated a relation to factor 3 and 4. While each item within every factor distinctly represents its respective construct, the participants’ comprehension of the questions within one factor appears to align with their understanding of questions from other factors. For example, the following statement in factor SE: you are confident that you will be able to get enough rest and exercise; factor SSA: you plan to practice adequate resting and exercise habits to prevent dementia; and factor SF: you have received assistance and support in practicing dementia prevention behaviors from family members, such as managing diet, taking medication, exercising, and brain exercises. From this example, participants may understand that all these questions are about rest and exercise. Hence, these findings suggest the need for modifying the questions in factors 1, 3, and 4 to emphasize the unique constructs represented by each factor. Consistent with our present study, Kim and colleagues also observed the association of factors within a model of phlegm pattern in a Korean population [42]. However, they suggest that while each factor may theoretically be independent, it cannot be entirely independent in real-world situations [42].

The DPSMBQ is the first questionnaire developed to specifically consider dementia as a complication of Type 2 Diabetes Mellitus (T2DM). However, the factors related to preventive behaviors for dementia in older adults with T2DM are similar to those assessed in self-care behavior questionnaires for T2DM, such as the Summary of Diabetes Self-Care Activities (SDSCA) [43,44] and the Diabetes Self-Management Assessment Report Tool (D-SMART) [45]. Regarding the DPSMBQ, a comprehensive review found that the initial version of the questionnaire included six factors related to dementia preventive behaviors, including (1) dietary habits, (2) non-smoking and alcohol-avoiding habits, (3) leisure and physical exercise habits, (4) stress management and brain exercise, (5) depressant prevention behaviors, and (6) drug adherence and follow-up habits; the EFA revealed that these factors were subsequently transformed into seven factors. These consisted of (1) RSM: relaxation and stress-management habits, (2) DAF: drug adherence and follow-up, (3) EX: exercise habits, (4) AFC: appropriate food consumption habits, (5) SAS: habits involving sweet beverages and adding seasoning, (6) FAC: fatty acid consumption habits, and (7) NSA: non-smoking and alcohol-avoiding habits. According to the new factors, two of these factors, including DAF and NSA, are similar to those identified in the literature review we conducted for the development stage. In contrast, the dietary habits factor, one of six factors for dementia preventive behaviors, was transformed into three new factors: AFC, SAS, and FAC. The reason for these results may be that the participants in EFA had a history of T2DM for 10.05 (SD = 7.61) years and, therefore, they may have previously received education from healthcare providers about how to control blood sugar levels through dietary choices in food categories [46,47,48].

For other factors, the physical exercise item from the leisure and physical exercise habits factor, and the brain exercise item from the stress management and brain exercise factor, were combined into the EX. This may be because older adults integrate their physical exercise and brain activity into exercises. Lastly, the leisure item from the leisure and physical exercise habits factor, the stress management item from the stress management and brain exercise factor, and depressant prevention were combined into the RSM factor. The combination of stress management and depression prevention might be because effective approaches to manage depression may be similar to those used for coping with stress [49,50].

Following the EFA, the CFA for the DPSMBQ was found to have degrees of freedom (χ^2^/df) < 2, indicating a superior fit between the hypothesized model and the sample data [51], but the RMSEA value of 0.057 only indicated an acceptable fit [34]. The CFI and TLI values of this sample, 0.898 and 0.882, respectively, are close to 0.900, which indicates a relatively good fit [52]. Based on these indices, this sample has a good fit overall. In addition, factor loading and CR should be equal to or greater than 0.707 for good convergent validity, but factor loadings greater than 0.4 or higher are acceptable [53]. Moreover, the 17 loadings are greater than 0.707 and 10 loadings are between 0.579 and 0.672 for items i9, i16, i18, i19, i20, i21, i23, i24, i26, and i27. All items in factors 5, 6, and 7 showed relatively low convergent validity. Similarly, the AVE of factors 5, 6, and 7 is lower than 0.500, resulting in these factors explaining more errors than the variance in their constructs. Nevertheless, all factors had a CR value greater than 0.707, and CR is found to most commonly represent convergent validity [54]. The results of convergent validity might have been affected by the comprehension of negative and positive questions in one questionnaire, particularly as comprehension can be age dependent [55]. In subsequent studies, we recommend modifying and re-evaluating aspects of this model, such as the SAS items (7, 8, 9, 11), FAC items (4, 13), and NSA items (14, 15, 16), which could be re-stated to underscore that these are positive behavioral traits, thereby reducing confusion in the older adults.

The DP-IFSM-PQ and DPSMBQ questionnaires provide a valuable psychoeducational tool for older adults with T2DM and their families to better understand self-management strategies aimed at preventing dementia, while also fostering the role of family members in caregiving. Furthermore, healthcare professionals, including nurses, can utilize these questionnaires to assess and monitor the dementia-preventative health behaviors of older adults with T2DM. The data gathered can inform the development of tailored interventions for this population, thereby enhancing the efficacy of dementia prevention efforts. The questionnaires could also be used as outcome measures for relevant psychosocial intervention studies, pending a test–retest reliability assessment.

Taken together, our study presents the psychometric properties of the newly developed Thai-language DP-IFSM-PQ and DPSMBQ, offering researchers a tool to identify dementia prevention behaviors. Furthermore, these questionnaires can be adapted to specific contexts and utilized in evaluating intervention programs for dementia prevention in older people with T2DM, which may involve family members. The questionnaires may also be useful for assessing and evaluating changes in dementia self-management behaviors within a clinical context.

This study has several methodological limitations that require consideration when interpreting the findings. The results are not representative of the entire older Thai population with T2DM, as study participants were recruited from six community hospitals in Chiang Mai (Northern Thailand). Therefore, the findings can only be generalized to participants located in that area. Future studies should aim to recruit older adults with T2DM from multiple sites located across the country to improve the generalizability of the findings. The survey response rate was also relatively low, which also casts some doubt on the representativeness of the sample. In addition, the items were generated by conducting a literature review, rather than stakeholder consultation, and we assessed face validity with a relatively small group of older adults with T2DM; both of which may negatively affect question comprehension, convergent validity, and discriminant validity. Therefore, future studies should consider a wider stakeholder group in the development of questionnaires, including T2DM patients, family members, nurses, and researchers. All data were also self-reported and may be subject to social desirability reporting biases, ability to interpret questions, and familiarity with rating scales more generally. Finally, this study did not evaluate test–retest reliability, because the follow-up period of the participants in the hospital was too long. Therefore, we do not know how reliable these questionnaires are over time. Subsequent studies should consider including a test–retest assessment within a suitable time frame.

## 5. Conclusions

The new Thai-language DP-IFSM-PQ and DPSMBQ questionnaires can be applied to Thai older adults with T2DM, since they show reasonable psychometric properties. Specifically, the study results show an acceptable-to-good fit, and Cronbach α indicates good internal consistency. Nevertheless, we recommend modifying some items and re-evaluating the psychometric properties in further studies with more diverse populations.

## Figures and Tables

**Table 1:** Demographic data of participants from EFA and CFA.

Demographics	EFA (n = 311)	CFA (n = 254)	Total (n = 565)
Gender, n (%)			
Male	117 (37.62)	88 (34.65)	205 (36.28)
Female	194 (62.38)	166 (65.35)	360 (63.72)
Mean Age (SD)	64.85 (2.77)	64.43 (2.83)	64.67 (2.81)
Marital Status, n (%)			
Single	17 (5.47)	8 (3.15)	25 (4.42)
Married	236 (75.88)	192 (75.59)	428 (75.75)
Widowed/Divorced/Separated	58 (18.65)	54 (21.26)	112 (19.82)
Educational Level, n (%)			
Illiterate	15 (4.82)	17 (6.69)	32 (5.66)
Primary School	221 (71.06)	186 (73.23)	407 (72.04)
Junior High School	37 (11.90)	21 (8.27)	58 (10.27)
Senior High School	21 (6.75)	20 (7.87)	41 (7.26)
Diploma/High Vocational Certificate	6 (1.93)	4 (1.57)	10 (1.77)
Bachelor’s degree or higher	11 (3.54)	6 (2.36)	17 (3.01)
Employment, n (%)			
Laborer	132 (42.44)	125 (49.21)	257 (45.49)
Trader	50 (16.08)	36 (14.17)	86 (15.22)
Government Officer	18 (5.79)	10 (3.94)	28 (4.96)
Unemployed	111 (35.69)	83 (32.68)	194 (34.34)
Mean Family Members (SD)	3.25 (1.38)	3.28 (1.41)	3.26 (1.39)
Family Structure, n (%)			
Husband and Wife	87 (27.97)	67 (26.38)	154 (27.26)
Single Family	94 (30.23)	72 (28.35)	166 (29.38)
Extended Family	130 (41.80)	115 (45.28)	245 (43.36)
Family member with T2DM, n (%)			
Yes	87 (27.97)	77 (30.31)	164 (29.03)
No	224 (72.03)	177 (69.69)	401 (70.97)
Mean Family Monthly Income in USD (SD)	314.16 (532.27)	267.30 (374.97)	293.09 (468.33)
Religion, n (%)			
Buddhist	303 (97.43)	248 (97.64)	551 (97.52)
Christian	8 (2.57)	6 (2.36)	14 (2.48)
Duration of T2DM	10.05 (7.61)	9.80 (6.97)	9.97 (7.36)
Mean HbA1C (SD)	7.67 (1.63)	7.64 (1.76)	7.66 (1.68)

HbA1C: glycated hemoglobin; T2DM: type 2 diabetes mellitus; USD: US dollars.

**Table 2:** The results from the EFA of the Dementia Preventive Individual and Family Self-Management Process Questionnaire (DP-IFSM-PQ).

Items	Component				Communality
	1 SSA	2 KB	3 SF	4 SE	
16	0.813				0.812
17	0.800				0.818
18	0.851				0.661
19	0.859				0.851
20	0.852				0.756
21	0.875				0.877
22	0.825				0.850
23	0.869				0.901
24	0.890				0.870
25	0.878				0.810
26	0.838				0.820
27	0.889				0.804
1		0.538			0.569
2		0.602			0.643
3		0.632			0.679
4		0.940			0.771
5		0.768			0.762
6		0.755			0.682
7		0.759			0.666
8		0.714			0.659
9		0.754			0.734
28			0.564		0.902
29			0.555		0.871
30			0.526		0.863
10				0.601	0.633
12				0.743	0.738
13				0.810	0.827
14				0.850	0.756
15				0.873	0.739
Sum of Squares	18.044	2.720	1.105	1.050	
% of Variance	60.147	9.067	3.684	3.500	
% Cumulative	60.147	69.214	75.898	76.398	
Cronbach’s α	0.925	0.927	0.872	0.855	

SSA: Self-regulation skills, and abilities to conduct dementia prevention behaviors; KB: the knowledge and beliefs of dementia prevention; SF: the social facilitation necessary to carry out dementia prevention behaviors; SE: the self-efficacy necessary to conduct dementia prevention behaviors. Extraction method: principal component analysis. Rotation Method: varimax.

**Table 3:** The results from the EFA of the Dementia Preventive Self-Management Behavior Questionnaire (DPSMBQ).

	Factor Loading							Communality
	1 RSM	2 DAF	3 EX	4 AFC	5 SAS	6 FAC	7 NSA	
19	0.722							0.623
20	0.790							0.728
21	0.816							0.762
23	0.785							0.740
24	0.808							0.729
25	0.826							0.721
26		0.725						0.659
27		0.781						0.745
28		0.708						0.601
29		0.781						0.743
17			0.874					0.860
18			0.869					0.777
22			0.817					0.826
1				0.555				0.642
2				0.506				0.574
3				0.692				0.596
5				0.669				0.643
7					0.645			0.503
8					0.747			0.597
9					0.679			0.582
11					0.547			0.633
4						0.722		0.581
6						0.566		0.492
13						0.771		0.611
14							0.738	0.623
15							0.816	0.711
16							0.675	0.580
Sum of Squares	4.926	2.670	2.522	2.497	2.293	2.148	1.910	
% of Variance	16.987	9.206	8.696	8.610	7.907	7.406	6.586	
% Cumulative	16.987	26.193	34.890	43.500	51.407	58.813	65.399	
Cronbach’s α	0.883	0.905	0.827	0.838	0.805	0.864	0.876	

RSM: relaxation and stress-management habits; DAF: drug adherence and follow-up; EX: exercise habits; AFC: appropriated food consumption habits; SAS: habits involving sweet beverages and adding seasoning; FAC: fatty acid consumption habits; NSA: non-smoking and alcohol-avoiding habits. Extraction method: principal component analysis. Rotation method: varimax.

**Table 4:** The correlation matrix, composite reliability (CR), and the average variance extraction (AVE) of the DP-IFSM-PQ.

Factor	(1)	(2)	(3)	(4)	CR
Factor 1	(0.456)				0.942
Factor 2	0.307 **	(0.474)			0.889
Factor 3	0.459 **	0.415 **	(0.658)		0.850
Factor 4	0.509 **	0.401 **	0.633 **	(0.578)	0.872
Mean	40.772	25.134	9.744	16.421	
S.D.	4.947	3.868	1.842	2.729	

Note: ** = *p* < 0.01.

**Table 5:** The correlation matrix, composite reliability (CR), and the average variance extraction (AVE) of the DPSMBQ.

Factor	(1)	(2)	(3)	(4)	(5)	(6)	(7)	CR
Factor 1	(0.629)							0.910
Factor 2	0.376 **	(0.559)						0.833
Factor 3	0.454 **	0.278 **	(0.703)					0.875
Factor 4	0.576 **	0.276 **	0.404 **	(0.539)				0.823
Factor 5	0.414 **	0.281 **	0.183 **	0.376 **	(0.381)			0.711
Factor 6	0.242 **	0.132 *	0.157 *	0.161 *	0.280 **	(0.492)		0.742
Factor 7	0.275 **	0.350 **	0.228 **	0.241 **	0.299 **	0.164 **	(0.458)	0.716
Mean	20.937	15.370	8.059	13.134	13.732	9.717	11.095	
S.D.	3.340	1.487	2.756	2.343	1.781	1.179	1.595	

Note: the values in the bracket represents the AVE, * = *p* < 0.05, ** = *p* < 0.01.

## Data Availability

The data presented in this study are available on request from the corresponding author due to not receiving prior approval from the ethics committee to deposit the raw data in a publicly accessible database.

## Supplementary Materials

The following supporting information can be downloaded at: https://www.mdpi.com/article/10.3390/nursrep14040277/s1, Table S1: The CVI of The Dementia Preventive Individual and Family Self-Management Process Questionnaire (DP-IFSM-PQ); Table S2: The CVI of the Dementia Preventive Self-Management Behavior Questionnaire (DPSMBQ).

## Public Involvement Statement

No public involvement in any aspect of this research.

## Guidelines and Standards Statement

In the present study, we followed EQUATOR guidelines for reporting observational research by using the STROBE checklist [56].
