# Supplementary material for: Dementia Prevention Self-Management in Older Thai Adults with Type 2 Diabetes: Development and Psychometric Properties of Two Questionnaires
Source: Nurs Rep. 2024 Dec 2;14(4):3786–802. doi: 10.3390/nursrep14040277 (PMC11677148; doi:10.3390/nursrep14040277)
Supplement: Supplementary file 1 [file nursrep-14-00277-s001.zip › nursrep-3167661-supplementary.pdf]

Table S1. The CVI of The Dementia Preventive Individual and Family Self-Management Process Questionnaire (DP-IFSM-PQ)

Statement in items	I-CVI
**The knowledge and beliefs of dementia prevention**	
Item 1. Older people with diabetes have a higher risk of developing dementia than older people without diabetes.	1
Item 2. Consuming a disease-specific diet that manages blood sugar levels can help prevent dementia.	1
Item 3. Avoiding smoking, exposure to cigarette smoke, and abstaining from alcoholic beverages can help prevent dementia.	1
Item 4. Adequate rest and regular exercise can help in preventing the onset of dementia.	1
Item 5. Appropriate stress management and brain exercise can prevent the development of dementia.	1
Item 6. Attending social events and socializing outdoors can prevent the development of dementia.	1
Item 7. Taking medication regularly and attending scheduled medical check-ups will help you manage diabetes well, thereby preventing the onset of dementia.	1
Item 8. Dementia is an incurable condition and tends to worsen gradually.	1
Item 9. People with severe dementia will experience memory loss and won't be able to help themselves.	1
**The knowledge and beliefs of dementia prevention**	
Item 10. You are confident that you will be able to consume disease-appropriate foods to control the accumulation of sugar levels.	1
Item 11. You are confident that you will be able to not smoke cigarettes and refrain from drinking alcoholic beverages	1
Item 12. You are confident that you will be able to get enough rest and exercise.	1
Item 13. You are confident that you can manage stress and maintain brain health.	1
Item 14. You are confident that you can participate in social activities and engage with others outside the home.	1
Item 15. You are confident that you can take your diabetes medication regularly and attend all scheduled check-ups.	1
**Self-regulation skills, and abilities to do dementia prevention behaviors**	
Item 16. You set a goal for practicing dementia-preventative behaviors.	1
Item 17. You observe yourself practicing dementia-preventive behaviors.	1
Item 18. You observe that you might start to develop dementia, such as forgetfulness, and therefore practice dementia prevention behaviors.	1
Item 19. You plan your dietary habits appropriate for diabetes to prevent dementia.	1

Statement in items	I-CVI
Item 20. You plan to practice smoking cessation and be able to not smoke cigarettes and refrain from drinking alcoholic beverages to prevent dementia.	1
Item 21. You plan to practice adequate resting and exercise habits to prevent dementia.	1
Item 22. You plan to attend social events, and socialize outside your home to prevent dementia	1
Item 23. You plan to practice stress-management behaviors and exercise your brain to prevent dementia.	1
Item 24. You plan to practice behavioral prevention of depression to prevent the development of dementia.	1
Item 25. You plan to practice proper medication habits and undergo regular check-ups as part of your plan to prevent dementia.	1
Item 26. You always evaluate the effectiveness of dementia prevention behaviors against the goals you set for yourself.	1
Item 27. If you find that you are unable to practice dementia prevention behaviors, you can consult your family, neighbours, and healthcare workers.	1
**Social facilitation to do dementia prevention behaviors**	
Item 28. You have received assistance and support in practicing dementia prevention behaviors from family members, such as managing diet, taking medication, exercising, and brain exercises.	1
**Social facilitation to do dementia prevention behaviors**	
Item 29. You have received encouragement from neighbours in practicing dementia prevention behaviors.	1
Item 30. You have received assistance and support from medical professionals in practicing dementia prevention behaviors, such as providing knowledge and advice on dementia prevention behaviors.	1
**S-CVI**	**1**

Table S2. The CVI of the Dementia Preventive Self-Management Behavior Questionnaire (DPSMBQ) 1

Statement in items	I-CVI
**Dietary habits**	
Item 1. You consume adequate portions from all five food groups (Carbohydrate, Protein, Lipid, Minerals, Vitamins), maintaining a balanced and proportionate diet.	1
Item 2. You adhere to regular mealtimes and ensure the consumption of all meals.	1
Item 3. You consume fish fillet, skimmed milk, and soy milk as part of your diet.	1
Item 4. You consume meats like beef and lean meats as part of your diet.	1
Item 5. You include nuts, green leafy vegetables, whole grains, and grains in your diet.	1
Item 6. You choose to use vegetable oils such as rice bran oil, soybean oil. in cooking	1
Item 7. You consume foods or beverages high in sugar content, such as ice cream, desserts, pastries, cookies, soft drinks, energy drinks, fruit juices, and coffee.	1
Item 8. You include additional sugar in your meals at every serving.	1
Item 9. You opt for artificial sugars or sweeteners as a substitute for natural sugar in your diet.	1
Item 10. You eat salty or high-sodium foods, such as ready-made food, processed food, and fermented food	1
Item 11. In the food eaten at each meal, you add seasonings such as salt/fish sauce/soy sauce/ketchup.	1
Item 12. You opt for vegetable oils like rice bran oil and soybean oil for cooking purposes.	1
Item 13. You consume foods high in cholesterol content, such as egg yolks, animal offal, animal brains, squid, oysters, and shrimp.	1
**Non-smoking and alcohol-avoiding habits**	
Item 14. You consume alcoholic beverages like beer, spirits, and herbal liquor.	1
Item 15. You smoke cigarettes or use tobacco.	1
Item 16. When encountering a person nearby smoking or in a smoked area, you prefer to walk away immediately.	1
**Leisure and physical exercise habits**	
Item 17. You have moderate-level exercises like walking, Chinese boxing, etc., consistently, for at least 30 minutes, 3-5 times a week, without taking breaks of more than 2 consecutive days.	1
Item 18. You exert or make physical movements such as digging the ground, doing housework, washing cars, walking fast, etc., until you feel somewhat tired or more tired than usual by breathing slightly faster than usual.	0.83
Item 19. You get an average of 6–8 hours of sleep per day without any signs of freshness, fatigue, or drowsiness.	1

Statement in items	I-CVI
**Stress management and brain exercise**	
Item 20. If you are angry or uncomfortable, you can control your emotions to calm down.	1
Item 21. If you are stressed, you can manage stress to reduce it.	1
Item 22. You do brain exercises such as playing games, doing math, meditating, and playing cards regularly.	1
**Depressant prevention behaviors**	
Item 23. You attend social events and meet others outside your home.	1
Item 24. You accept your strengths and weaknesses.	1
Item 25. When you feel sad, disappointed, or sad you have a way of expressing your feelings that is appropriate for yourself.	1
**Drug adherence and follow-up habits**	
Item 26. You always take medication for diabetes and other medical conditions according to your doctor's treatment plan. Without increasing or decreasing the dose on their own.	1
Item 27. You borrow diabetes medication from others to take.	1
Item 28. You forget to bring your diabetes medication when traveling on provincial errands.	1
Item 29. You go to see the doctor for every appointment.	1
**S-CVI**	**0.97**

* Negative behaviors (item 4, 7-15, 27-28)
